# ‘Doing the right thing’: factors influencing GP prescribing of antidepressants and prescribed doses

**DOI:** 10.1186/s12875-017-0643-z

**Published:** 2017-06-17

**Authors:** Chris F. Johnson, Brian Williams, Stephen A. MacGillivray, Nadine J. Dougall, Margaret Maxwell

**Affiliations:** 1 Pharmacy and Prescribing Support Unit, NHS Greater Glasgow and Clyde, 2nd Floor, Main Building, West Glasgow Ambulatory Care Hospital, Dalnair Street, Yorkhill, Glasgow, G3 8SJ UK; 2 School of Health and Social Care, Edinburgh Napier University, Sighthill Court, Edinburgh, EH11 4BN UK; 3 School of Nursing and Health Sciences, University of Dundee, Airlie Place, Dundee, DD1 4HN UK; 4 Nursing Midwifery and Allied Health Professionals Research Unit, University of Stirling, Unit 13 Scion House, Stirling University Innovation Park, Stirling, FK9 4NF UK

**Keywords:** General practice, Primary health care, Depression, Antidepressive agents, Doctor-patient relationship, Qualitative research

## Abstract

**Background:**

Antidepressant prescribing continues to increase, with 5-16% of adults receiving antidepressants annually. Total prescribing growth is due in part to increased long-term use, greater selective serotonin re-uptake inhibitor (SSRI) use and the use of higher SSRI doses. Evidence does not support routine use of higher SSRI doses for depression treatment, and factors influencing the use of such doses are not well known. The aim of this study was to explore factors influencing GPs’ use of antidepressants and their doses to treat depression.

**Methods:**

Semi-structured interviews with a purposive sample of 28 practising GPs; sampled by antidepressant prescribing volume, practice size and deprivation level. A topic guide drawing on past literature was used with enough flexibility to allow additional themes to emerge. Interviews were audio-recorded and transcribed verbatim. Framework analysis was employed. Constant comparison and disconfirmation were carried out across transcripts, with data collection being interspersed with analysis by three researchers. The thematic framework was then systematically applied to the data and conceptualised into an overarching explanatory model.

**Results:**

Depression treatment involved ethical and professional imperatives of ‘doing the right thing’ for individuals by striving to achieve the ‘right care fit’. This involved medicalised and non-medicalised patient-centred approaches. Factors influencing antidepressant prescribing and doses varied over time from first presentation, to antidepressant initiation and longer-term treatment. When faced with distressed patients showing symptoms of moderate to severe depression GPs were confident prescribing SSRIs which they considered as safe and effective medicines, and ethically and professionally appropriate.

Many GPs were unaware that higher doses lacked greater efficacy and onset of action occurred within 1-2 weeks, preferring to wait 8-12 weeks before increasing or switching. Ongoing pressures to maintain prescribing (e.g. fear of depression recurrence), few perceived continuation problems (e.g. lack of safety concerns) and lack of proactive medication review (e.g. patients only present in crisis), all combine to further drive antidepressant prescribing growth over time.

**Conclusions:**

GPs strive to ‘do the right thing’ to help people. Antidepressants are only a single facet of depression treatment. However, increased awareness of drug limitations and regular proactive reviews may help optimise care.

**Electronic supplementary material:**

The online version of this article (doi:10.1186/s12875-017-0643-z) contains supplementary material, which is available to authorized users.

## Background

Antidepressant prescribing has increased substantially across Western societies over the last 40 years [1–3], attracting much discussion, debate, concern and controversy [1, 4, 5]. The issues are wide and varied: whether antidepressants are overprescribed or not [4, 5]; whether antidepressants are effective or ineffective in treating depression [6]; Westernised societies’ expected right to happiness and consequential medicalisation of unhappiness [7, 8]; the direct/indirect role which the pharmaceutical industry may have in influencing the definition of depressive disorder [9, 10]; promoting simple marketing models of depressive illness [11]; ‘ghostwriting’ antidepressant studies [9, 10]; and more controversially, reporting bias, missing data [12], and antidepressant associated self-harm [13]. Despite these debates and controversies, depression remains a common, recurrent, debilitating and potentially fatal condition with antidepressants being only one of the many components of depression treatment which have been shown to be effective [14–17].

Although prescribing continues to increase, there has been no corresponding increase in depression incidence or prevalence [18, 19]. Most antidepressants are prescribed by general practitioners (GPs) with the majority (60-85%) of adults receiving them for the treatment of depression [20–22] and a minority receiving them for other conditions [23], with an estimated 5-16% of adults receiving antidepressants in Europe and USA annually [24, 25]. Factors that have contributed to prescribing growth include: the introduction of tricyclic antidepressants (TCAs) in the late 1960s [1], then the addition of selective serotonin re-uptake inhibitors (SSRIs) in the 1980s [26]; increased long-term prescribing in the 1990s and 2000s [19]; lack of regular review [27]; and more recently the use of higher doses [21, 28, 29].

SSRIs are of particular interest as they account for the majority of antidepressant prescriptions, dominating antidepressant volumes across Western Societies [24, 25], and are routinely prescribed at higher doses than previously reported in the literature in the UK and New Zealand [21, 29]. In one study, higher than standard SSRI doses were associated with the practice which the patient attended; long-term use of SSRIs; and co-prescribing of SSRI with long-term benzodiazepines and/or z-hypnotics (B&Zs) [22]. However, current evidence does not support the routine use of higher licensed doses as they lack greater efficacy [17, 30, 31]. Higher doses are also associated with more adverse effects including anxiety, agitation and insomnia [30] which may lead to co-prescribing of sedating drugs such as B&Zs, antipsychotics and other antidepressants such as trazodone. Such combinations of high dose SSRI and sedating antidepressant use may also influence the large variations in antidepressant volumes prescribed between practices [32]. There is a need to understand these variations in prescribing practice.

## Methods

### Aim

The aim of this study was to explore factors influencing GPs’ use of antidepressants and their doses to treat depression.

### Design and setting

Quantitative methods can be limited in providing depth of clarity on this issue, therefore qualitative methods were used to enable an in-depth understanding of GPs’ perspectives and rationale for prescribing. Focus groups were considered inappropriate as it was expected that predominant voices may drown out more moderate voices, and impractical for busy GPs to attend within normal working hours. Therefore we captured in-depth GP perspectives on their practical experience and opinions of what influences their antidepressant prescribing through one-to-one, face-to-face, interviews conducted in each GP’s office.

### Sampling and recruitment

We recruited 28 GPs working in general practices across the National Health Service Greater Glasgow and Clyde (NHSGG&C) region in Scotland. NHSGG&C provides taxpayer funded healthcare services for a diverse population of 1.2 million people across a varied urban region containing 260 general practices.

In order to achieve information-rich cases and diverse perspectives, a purposive sample was sought by allocating all general practices in NHSGG&C to a sampling frame using practice characteristics: volume of antidepressants prescribed; number of GPs; and practice deprivation score. We aimed to recruit 30 GPs from across NHSGG&C with an equal number of male and female GPs, and a proportion of GPs partners working in the same practice (7 practices).

Antidepressant prescribing data for the 260 practices was obtained from the Prescribing and Information System for Scotland for year to March 2014, as previously described [22]. The 260 practices were first ranked from lowest to highest antidepressant prescribers, and then categorised as low, medium and high prescribers based on the number of defined daily doses (DDDs)/1000 patients. These were then subcategorised as small (single handed GP), medium (2-3 GPs) and large (≥4 GPs) by the number of GPs contracted to individual practices, as recorded in April 2014 [33]. Finally, practices were ranked by Scottish Index of Multiple Deprivation rank derived from each practice’s postcode [34], and categorised as being in areas of low, medium and high deprivation.

Between August 2014 and December 2015, GPs matching the sampling frame were initially contacted by letter and followed up by telephone within 2 weeks by administrative staff. In total 188 GPs were written to, in groups of 30 to 40, during the study period. GPs were informed about the aims of the study, that study findings would be used to support appropriate antidepressant use within NHSGG&C, and that previous NHSGG&C work had identified the routine use of higher SSRI doses [21, 22]. Potential participants showing interest were then contacted by CFJ to arrange interviews at their offices’. Prior to interviews starting, CFJ discussed the study and sought participant’s consent for inclusion in the study. GPs were not incentivised to participate in any way. A total of 28 GPs participated, 14 male and 14 female, see Table 1.

**Table 1:** Individual GP and practice characteristics

Individual GP Characteristics (*n* = 28)			
Female (%)			14 (50)
Median age (range)			43 (33 to 60)
Years since qualified as doctor, median (range)			19 (10 to 37)
Years as a GP median (range)			12 (2 to 33)
Number of GPs with psychiatry training as part of GP training rotation (%)			19 (68)
Number of GPs with extra psychiatric training/experience as locum or psychiatry trainee (%)			4 (14)
Individual practice characteristics (*n* = 20)			
Antidepressant volumes in DDD/1000 patients, n (GPs)	Low = 9 (10)	Medium = 4 (6)	High = 7 (12)
Number of GP partners, n (GPs)	Small (single handed) = 1 (1)	Medium (2-3 GPs) = 10 (13)	Large (≥4 GPs) = 9 (14)
Deprivation, n (GPs)	Low = 6 (6)	Medium = 5 (7)	High = 9 (15)
Training practice, n (GPs)	Yes = 10 (16)	No = 10 (12)	

*DDD* defined daily doses. DDDs are units of measurement defined by the World Health Organization as ‘the assumed average maintenance dose per day for a drug used for its main indication in adults’. DDDs do not necessarily reflect the recommended or prescribed daily dose but allow a convenient method to compare prescribing volumes between organisations [59]

### Data collection

One-to-one, face-to-face, semi-structured interviews were carried out by one researcher, a prescribing support pharmacist (CFJ), at the GP’s office. Interviews lasted between 15 and 55 min with most lasting approximately 30 min. Although an initial topic guide was developed using previous literature, it was flexible enough to allow additional topics to emerge (see Additional file 1: Topic guide for first interview (v6) and final version of topic guide (v14)). Interviewees were also asked for their perspectives on factors associated with higher SSRI dose use for depression treatment as previously identified [22]. Early interviews and subsequent interviews informed the refinement of subsequent topic guides and the inclusion of emergent themes expressed as GPs’ views and experiences at interview. Interviews were audio-recorded and transcribed verbatim.

### Analysis

Data analysis was inductive and continuous, and began from the start of data collection. Constant-comparative technique was used [35] while adopting Framework analysis [36] as the broad structure for the analytical procedure. Familiarisation with, and constant-comparison between transcripts permitted additional important themes to emerge. Transcripts were initially read and emerging themes and patterns discussed by three authors (CFJ, BW and MM) to identify a thematic framework. Constant-comparison and disconfirmation were then carried out repeatedly and systematically (by CFJ) between transcripts to enhance and confirm the thematic framework. Data collection was interspersed with analysis, with findings informing subsequent interviews, allowing interviewees to explain and/or expand on key themes. As CFJ was aware that being a prescribing support pharmacist may influence GPs’ responses, interviewees were asked if this could have affected their responses. The thematic framework was then systematically applied to the data which were indexed and summarised in charts, by CFJ. Broad themes and patterns (mapping), both within and across interviews were identified. NVivo 11 was used to store and organise data.

From this process, an understanding of the factors influencing GPs decision-making regarding the use of antidepressants and their doses emerged, and were conceptualised into an overarching explanatory model of factors influencing prescribing.

### Ethical approval

The study was approved by the Ethics Committee of the School of Health Sciences, University of Stirling 21st April 2014.

## Results

**Fig. 1:**
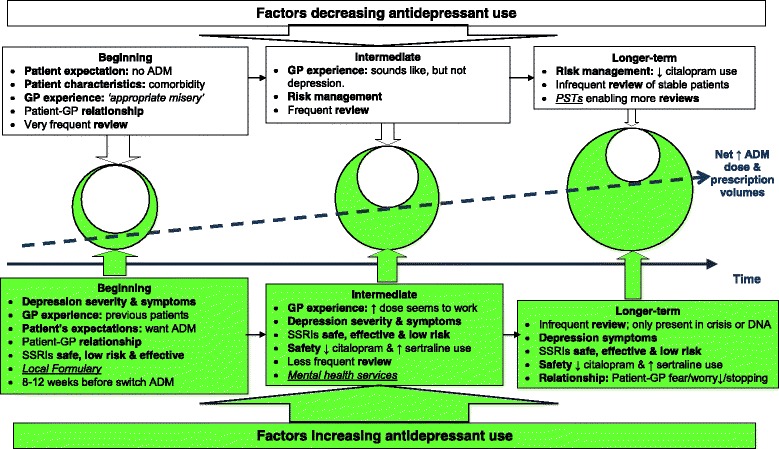
Factors increasing and decreasing antidepressant prescribing for depression treatment. Factors strongly influencing antidepressant prescribing bold and moderately influential factors in italics and underlined. ADM: antidepressant. DNA: do not attend. PST: Prescribing Support Teams. SSRI: selective serotonin re-uptake inhibitor

Analysis revealed that depression treatment involved two key overarching concepts of ‘doing the right thing’ and achieving the ‘right care fit’ for individuals. This involved medicalised and non-medicalised patient-centred approaches with antidepressants only being a single facet of treatment. However, factors influencing antidepressant prescribing and prescribed doses varied over time from first presentation and the beginning of treatment, to antidepressant initiation and longer-term treatment (Fig. 1). Seven interwoven factors influenced antidepressant prescribing. Five of these factors can be described as strongly influential: 1) Depression diagnosis and management; 2) Patients’ expectations and characteristics; 3) GP’s experience and relationships; 4) Antidepressant use: safety, risk management and effectiveness; 5) Review frequency. Two were moderately influential: 6) Local prescribing resources and 7) Mental health services. As outlined below with illustrative quotes, also see Additional file 2: Supplementary quotes.

The magnitude of the influence exerted by these factors varied with time. For example, review frequency was high and highly influential at the beginning of treatment in supporting and increasing appropriate antidepressant use for more severe illness, whereas in the intermediate period following the first few months of antidepressant treatment, review frequency decreased, thereby reducing the potential for appropriate continuation or discontinuation. In the longer-term, reviews became more infrequent and coupled with some patients presenting in crisis further increased cumulative prescribing (Fig. 1).

When faced with patients showing symptoms of moderate to severe depression, GPs were confident prescribing SSRIs, which they considered safe and effective medicines. Prescribing was seen ethically and professionally as ‘doing the right thing’ for the patient in the face of distress. However, many were unaware higher doses lacked greater efficacy and onset of action occurred within 1-2 weeks, preferring to wait 8-12 weeks before increasing or switching. The ongoing pressures to maintain prescribing (e.g. patient wishes, fear of depression recurrence), few perceived continuation problems (e.g. lack of safety concerns) and the lack of proactive medication reviews (e.g. patients only present in crisis) contributed to further antidepressant prescribing growth over time (Fig. 1).

### Depression diagnosis and management

#### Diagnosing depression

**Fig. 2:**
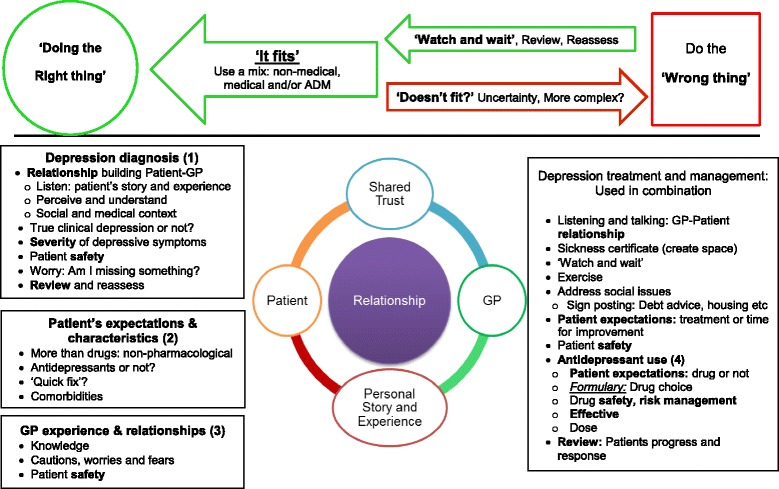
Balancing treatment and management options. Factors strongly influencing antidepressant prescribing bold and moderately influential factors are in italics and underlined

Across the sampling frame diagnosing depression was rarely seen as a simple task or process. This was due to a variety of issues and complex interactions involving: normal life events; relationship problems; social and environmental issues; patients’ expectations of happiness; duration of symptoms; mixed anxiety and depressive symptoms; comorbidities; and the risk of medicalising normal and *“appropriate misery” (D21,18p3)* due to life events. However, time well spent in this initial presentation period – regularly reviewing patients – was seen as an important part of the biopsychosocial assessment. Some practitioners routinely used depression rating scales to quantify depression severity or as a tool to support discussions with patients, whereas others found rating scales hampered assessment, or were not used as they lacked a social domain. Patient information was used to ascertain and balance how well signs, symptoms and subjective assessment fitted with standardised concepts of clinical depression and severity to achieve the best treatment ‘fit’ (Fig. 2).

*…the bottom line for GPs is that we want to help, we want to offer something that we think will help. D12,8*

*You also want to look at the person as a whole and find out where they are in their life. You have to assess the actual severity of the situation before determining what kind of treatment would be appropriate for them. Then, we would go down the route of discussing what sort of therapies we could offer them. D25,6*

In general, GPs rarely prescribed antidepressants at the first presentation, unless patients had a recurrent depressive episode where antidepressants were previously effective, as a large proportion of patients presented in crisis and were experiencing an acute reaction to life events or stressors. GPs in our study viewed it as more important to listen to patients and discuss issues in the first instance, especially for mild to moderate forms of depression where patients needed someone to talk to, not prescribe. Allowing a period of ‘watch and wait’ where depressive symptoms would remit.

*…for a lot of people with a mild to moderate depressive illness, is to say, “you might not need anything here. You might just need, someone to talk to you about it and some support and things might improve on their own.” D12,96*

Although for some patients, there was an expectation about receiving something.

*They think they’re coming here [pause] for me to do something for them [empathetically said]. And that, they almost feel as if there needs to be a physical display of that, like the prescription or whatever. D2,14*

For more severe cases, and for patients that GPs knew well, they would consider prescribing at the first presentation if symptoms were sufficiently severe to warrant an antidepressant. However, this was not routine practice. Referral to specialist Community Mental Health Teams (CMHTs) was also considered for people with more severe symptoms.

#### More than drugs

Treatment involved more than drugs. As already identified, GPs considered listening, talking and allowing patients time for spontaneous remission as an important core part of appropriate care, treatment and management. For all severities of depression, and where patients preferred not to take antidepressants for moderate to more severe depression due to stigma or personal choice, GP’s embraced, supported and used multiple options to manage and treat signs and symptoms in line with the patient’s preferences. This included a varied array of medical and non-medicalised approaches: creating space for patients by using sickness certification; exercise and exercise referrals to local council gyms; counselling; signposting to information sources e.g. Links-workers to address money worries; bibliotherapy in libraries; online cognitive behavioural therapy e.g. Moodgym; talking therapies via NHS and non-NHS providers in combination with or without antidepressants. A multifactorial interwoven approach was thus created in achieving the ‘right care fit’ and ‘doing the right thing’, with drug treatment being only one of many therapeutic approaches.

*I explain to them that, “You have to look at this [responding to an antidepressant] in conjunction with other things.” So, it’s always going to be a multifactorial approach. It’s never going to be just one thing [an antidepressant]. D28,28.*

### Patients’ expectations and characteristics

Data indicated that GPs’ prescribing was not overtly influenced by patients’ expectations of receiving an antidepressant. The need for a clear benefit to the patient was still the main influence, assessed on the basis of knowing the patient’s history, comorbidities and social context. However, it was acknowledged that time pressures could play a role, as it was difficult to discuss and encourage the use of non-antidepressant alternatives if clinics were running late.

*I think most of my colleagues here wouldn’t prescribe unless they felt somebody was going to get benefit from them [antidepressants]. We all kind of have roughly the same sense of what’s bad and what’s good. D4,24*

Many GPs felt that some patients were looking for ‘a quick fix’, and that this was rooted in wider societal expectations that problems could and should be solved medically. However, it was usually those with milder symptoms that had greater expectations of ‘a quick fix’. As they presented in crisis there was an expectation to do something to solve the problem; a physical display with a prescription, which was sometimes driven by family members more than patients. For some the ‘quick fix’ was short-term antidepressant use, which stopped within a couple of months of starting, while others did not expect ‘a quick fix’ and wanted to avoid antidepressants altogether.

*I think there’s an expectation generally, that if there is a problem perhaps you know there is a pill for it. I think that is an expectation that’s held by a lot of people. Other people are very resistant to the idea of taking antidepressants. D18,6*

### GP experience and relationships

Experiential learning significantly influenced how GPs prescribed antidepressants. This cumulative knowledge was gained through a mixture of formal training, such as general practice training schemes and acute psychiatric experience, and informal reflective practice - seeing improvements in one patient and repeating the same intervention with others. However, with time and greater experience prescribers formulated their own ideas about depression management, becoming more *“idiosyncratic”* to achieve the ‘best care fit’ such as using mirtazapine rather than fluoxetine, where insomnia was a significant issue, or sertraline instead of citalopram for patients with cardiac disease.

*I think initially absolutely with guidelines and I guess, as I alluded to before, the more experienced I’ve got, the more idiosyncratic I’ve got. It tends to be how a patient’s presenting, so it might be side-effects or likely side-effects or beneficial side-effects that may guide me on where I would go [with treatment]. D24,20*

National and local guidelines were considered by GPs to weakly influence antidepressant prescribing, with some specialist resources being helpful in specific situations e.g. switching drugs. However, local prescribing resources, namely the formulary and prescribing support teams, did influence drug choices and cost effective prescribing decisions.

*We’ve got our in-house pharmacist, and it’s fantastic, ‘cause we sit down and talk about these things... For example, with venlafaxine slow release people, we’ve changed all of them [to lower cost ordinary release], and we resisted pressure from secondary care and patients as well. So that’s definitely a positive influence. Because we’re not pharmacists, and we don’t know nearly as much about pharmacology as pharmacists do. D26,129*

Most GPs indicated they prescribed within formulary guidance whereas psychiatrists and other specialists tended to prescribe third or fourth line agents which were outwith formulary guidance. This sometimes caused friction, especially with children and adolescents where the evidence is weaker; there were potentially greater safety concerns and risks; and shared care structures were lacking or not considered robust enough, thus providing a ‘poor care fit’ and raising potential medico-legal issues.

*The other issue is prescribing antidepressants in young people. We won’t prescribe antidepressants that are unlicensed in young people. We won’t prescribe them in people under the age of 18 because there is no shared-care protocol. Unfortunately, without the support of shared-care protocol we don’t feel really we have the specialist knowledge to be prescribing it much for [children and adolescents]. D25,29 We’ve been asked to prescribe sertraline and fluoxetine, I think, in people around about age 15. Both of which we’ve refused. We’ve refused all of them. The issue then is that they feel that once they’ve initiated it we should take over the prescription. But because there’s no shared-care protocol it still leaves us fairly vulnerable. So, we have still decided as a partnership that we won’t be involved in that…D25,31*

However, these frictions were partly overcome where there was good communication, supportive structures and good relationships.

Pharmaceutical companies were considered not to influence prescribing as GPs avoided seeing company representatives for a variety of reasons e.g. anger about ‘me-too’ drugs, promotion of active isomers of cheaper older drugs sold at a premium price. However, GPs acknowledged that companies had subtle influences on depression management.

*Well... escitalopram really pissed me off, I hate that sort of carry on, it was like loratadine and desloratadine, I just hate that! I mean ‘me-too’ drugs that happen to appear just as patents are running out and are another way of creaming money out of the unsuspecting public,… D21,70*

*…there was the Defeat Depression campaign and that was the Royal College. But the Royal College and GPs really got into tow I think with pharma in a big way, and I think actually that was probably fairly influential but,… pharma were probably being very very clever there, and more subtle than usual. I would say…people get quite well develop antibodies to pharma now. So they actually probably have to work harder to convince me… But they are more subtle, and they have subtle links. D18,23*

In general the media was considered not to influence prescribing, but some GPs were aware of previous media articles regarding fluoxetine and adolescent suicide, which had changed prescribing habits. The media was thought to influence patients’ attitudes and expectations regarding depression treatment, although this was often presented in a confused ambiguous manner.

*I think that the media give quite a muddled view on things. They all seem to be reporting the celebrities who are getting treatment or counselling for this, that and the other. And, then, on the other hand, they bash GPs for overprescribing antidepressants like sweeties.D3,20*

### Antidepressant use: safety, risk management and effectiveness

#### Drug choice

Across the sampling frame prescribing was influenced by GP’s prior clinical experience, severity of patient’s depressive symptoms and needs, along with age and comorbidities. Treatment options were agreed through GP-patient discussions.

*You aim to certainly do it [prescribe] in partnership with the patient. At the end of the day, if you don’t do it in partnership with them and you prescribed it, then they won’t take it anyway, so you do it in partnership with the patient. …based on advice, guidelines. I think there is an element of doing what you believe is the right thing from your own experience. D8,22*

SSRIs were seen as being effective, well tolerated and a safe choice, especially when compared to TCAs. Consideration was also given to the slight differences between SSRIs with fluoxetine seen by some as more stimulating and appropriate for depression, whereas sertraline and citalopram were considered more appropriate for mixed depression anxiety symptoms and better tolerated. The Medicines and Healthcare products Regulatory Agency (MHRA) safety warning regarding citalopram and escitalopram causing dose dependent QT interval prolongation, which is associated with ventricular tachycardia and sudden cardiac death [37], had influenced prescribers who were now using less citalopram and more sertraline.

*They’re safer. So, no one in their right mind now is going to give an MAOI if you’re a GP. There’s no good reason to start a tricyclic rather than an SSRI unless you’d been through a few of them [antidepressants] already. You know, there’s far less risk from a GP’s point of view in terms of overdosing, in terms of side effects from the medication. D22,33*

Mirtazapine’s side effects were considered beneficial for some patients, with weight gain being positive for underweight patients while the sedative effects alleviated insomnia and anxiety symptoms for some. Low dose mirtazapine was being used in preference to more traditional low dose trazodone or as a safer non-addictive alternative to avoid B&Zs.

*…well I know that it’s a funny drug [mirtazapine], because it’s supposed to be only sedating at low dose, because it has the antihistamine effect. We use it a lot at 15mg just for the sedating effects, as a non-addictive sleeping pill, really. D26,39*

Opinion was split when using mirtazapine to treat depression - some quickly increased to therapeutic doses while others maintained people on 15 mg subtherapeutic doses. In part this may have been influenced by CMHTs and Addictions Teams use of low dose mirtazapine as a single agent or in combination with other antidepressants. A small minority of GPs acknowledged that they rarely added another antidepressant to augment current treatment, e.g. adding mirtazapine to an SSRI, and considered their practice to be influenced by CMHTs as only a minority of these GPs had extensive psychiatric training. Others, however, questioned the appropriateness of combining antidepressants without specialist input and considered it as the specialist’s domain, as with other psychotropics e.g. antipsychotic augmentation with quetiapine. Although, most were comfortable initiating low dose amitriptyline for neuropathic pain for patients already receiving an antidepressant for depression.

*Well that’s one of the places I have been influenced by secondary care. Because a lot of the psychiatrists say, we’re going to add this to augment the effect of this. It’s usually mirtazapine and citalopram together. And I actually do think that works, I’m not quite sure the biochemistry behind that. But... erm, I now do that sometimes myself. It’s often for the poor sleepers. D26,65*

#### SSRI efficacy: time to effect and dose response

All GPs reported that they prescribed standard therapeutic SSRI doses: 20 mg daily for citalopram, fluoxetine and paroxetine, or 50 mg daily for sertraline. Half considered that SSRIs were effective within 2 to 4 weeks, with some indicating that some patients respond well within the first 2 weeks of treatment. The remaining half considered efficacy was achieved within 6 to 8 weeks. When SSRIs taken at therapeutic doses were ineffective or partially effective, a large proportion of GPs would wait 8 to 12 weeks before increasing the dose or changing antidepressants. Most of these prescribers were female and had completed GP psychiatric or extra psychiatry training but did not differ in other characteristics to GPs that increased or changed sooner. In part, persevering with one antidepressant for a longer period may have been due to concerns about giving people an adequate trial and fears of running out of pharmacological treatment options.

*Keep them on... probably quite some time, 2 or 3 months, and if they weren’t responding, then change. D17, 50*

From experience, a minority of GPs thought that standard SSRI doses provided maximum efficacy, with higher doses lacking greater benefits, so rarely increased or ‘pushed doses’ up. In contrast the majority considered higher SSRI doses were more efficacious with sertraline being routinely increased. There were no differences in characteristics between the two groups, with both acknowledging that psychiatrists routinely ‘pushed the dose’ of SSRIs. However when discussed as part of the MHRA advice restricting citalopram to lower doses, most observed that a few patients’ depressive symptoms worsened while most remained well controlled on lower doses.

*As we know the response to higher doses doesn’t grow, you know, parallel to the increasing dose. So, if we get a good response to the first dose... doubling the dose to, a higher dose doesn’t always make a big difference. That’s our clinical experience. D6,44*

Some prescribers acknowledged being drawn into responding to a patient’s distress by ‘doing something’ although they were aware the intervention may have limited or negligible benefits. Some considered this to be less than an ideal care ‘fit’ even though it provided patients with hope, demonstrated that patients had been listened to, and that all options were being tried.

*…when you’ve got a patient, a desperate patient in front of you wanting something to be done, it’s the temptation is to crank up the dose. Again, one of my colleagues will go up to much higher doses of fluoxetine than, than perhaps the rest of us would. D3,60*

#### SSRI efficacy: use of higher SSRI doses for depression treatment

GPs’ opinions were sought regarding previous observations that higher SSRI doses were routinely being prescribed [21, 28, 29] and that higher than standard SSRI doses for the treatment of depression were associated with the practice the patient attended, long-term use (receiving the same antidepressant for ≥2 years), and co-prescribing of long-term (≥8 weeks) B&Zs [22].

GPs considered that practice factors associated with higher SSRI doses may be due to more aggressive prescribers ‘pushing the dose’, but was in part due to prescriber’s experience, what worked with previous patients and/or if GPs had psychiatry training. Although GPs admitted to being more comfortable prescribing antidepressants and patients were more comfortable taking antidepressants, most practitioners considered that their prescribing was similar to their colleagues. Only two GPs considered that they prescribed more, one due to being female and seeing more female patients and the other because he prescribed lots of everything.

A few GPs highlighted differences in management styles between them and their colleagues relating to: frequency of review and follow up, use of alternatives, and again that a minority were happier prescribing antidepressant combinations for depression, whereas the majority were not.

*I think there’s two of us in the practice seeing more people who have got psychological problems. I would then however suspect that others might prescribe more antidepressants per head if you know what I mean. Whereas I would be more interested in trying alternatives to antidepressants. D18,26*

Higher doses associated with long-term SSRI use were considered to be due to a combination of factors, greater depression severity with more refractory symptoms and dose escalation over the years for those prescribed SSRIs in response to crises, as previous dose increases were considered effective. However, the dose may not have been reviewed and reduced at a later date, and then further increased with subsequent crises, with colleagues within the practice not feeling comfortable reducing and/or stopping medication because they had not increased the dose. As patients presented in crisis, and not when they are well, there were challenges in ensuring proactive routine antidepressant reviews and opportunities to appropriately reduce prescribing. GPs did however acknowledge that most patients who were proactively reviewed due to the MHRA citalopram warning were able to reduce or stop citalopram without any significant problems.

Patient and GP fears of relapse due to reducing or stopping antidepressants – causing more harm than good – were also discussed by some prescribers. Especially for patients with chronic depression, creating challenges for restarting, optimising and stabilising individuals.

*I suppose if you’ve got somebody that goes through crisis and they’re on a drug anyway for a long time, every time they have a crisis the dose might be bumped up and then not reduced. So, I wonder if there’s an element of just not reducing the drug when it’s appropriate... and patients psychologically seem to be quite dependent on these drugs as well. So, they might want that increased dose too. D10,93*

Higher SSRI doses associated with long-term B&Z treatment were considered to be linked with patients being more complicated, possibly having greater psychiatric multimorbidity as well as underlying social and personal issues not being addressed but medicated instead. Patients were generally more willing to engage and seek pharmacological treatment and resisted reductions. However, clinicians rarely consider adverse effects with higher SSRI doses increasing anxiety or B&Z’s lowering mood.

*…it is probably if you’ve got somebody who’s on long-term benzos it suggests they’re not very well. There’s long-term issues there. …probably most of those issues are not going to be dealt with by a drug. D24,74*

Finally, in summarising the factors influencing antidepressant prescribing and dose used, data indicated that ‘doing the right thing’ and appropriately initiating antidepressants where there was a clear need increased antidepressant prescribing growth, Fig. 1. However, after patients were established on antidepressants and with increasing treatment duration, there were fewer and fewer factors over time which provided counterbalances to reduce prescribing and use, thus explaining the phenomenon sustaining and driving net antidepressant prescribing growth over time.

## Discussion

### Summary

Diagnosising and treating depression was rarely seen as a simple task or process by GPs due to the complex interaction of normal life events, relationships, social and environmental pressures. Treatment involved ethical and professional imperatives expressed as two overarching concepts of ‘doing the right thing’ and achieving the ‘right care fit’ for individuals. This involved medicalised and non-medicalised patient-centred approaches, with antidepressants only being a single facet of individualised care (Fig. 2).

Five factors were strongly influential: 1) Depression diagnosis and management; 2) Patients’ expectations and characteristics; 3) GP’s experience and relationships; 4) Antidepressant use: safety, risk management and effectiveness; and 5) Review frequency. The magnitude of the influence exerted by these factors varied with the individual’s needs; treatment options; antidepressants prescribed and doses used over time, from first presentation, to antidepressant initiation and longer-term treatment. Many GPs were unaware that onset of action occurred within 1-2 weeks, preferring to wait 8-12 weeks before increasing or switching.

When faced with distressed patients showing symptoms of moderate to severe depression, GPs were confident prescribing SSRIs which they considered as safe and effective medicines, as well as ethically and professionally appropriate. However, individual patient comorbidities, national safety warnings and use of sedating antidepressants as non-addictive alternatives to B&Zs influenced drug choice for managing risks, optimising patient safety and benefit.

Many GPs were also unaware that higher SSRI doses lacked greater efficacy. This lack of awareness was accompanied by: ongoing pressures to maintain prescribing (e.g. patient wishes, fear of depression recurrence); few perceiving continuation as problematic (e.g. lack of safety concerns); and the lack of proactive medication reviews (e.g. patients only present in crisis), all combining to further drive antidepressant prescribing growth over time (Fig. 1).

### Strengths and limitations

Strengths: The main strength of this study was the sampling frame used which ensured the views of a wide variety of working GPs’ experiences were captured from a large urban region with the same formulary, prescribing support team and local depression guidelines. This study also sought local GP perspectives on previous research findings from other general practices within their urban region [22], thereby allowing GPs to use their unique insight in considering local and national contextual issues contributing to the use of higher SSRI doses, as seen elsewhere [28, 29]. Another advantage was that during the study period there were no changes to the local formulary, prescribing support team activities or depression guidelines, although in May 2015 the British Association of Psychopharmacology issued new guidelines [17]. This was assessed as having negligible effects on GPs’ responses, as most acknowledged guidance was a weak influencing factor on prescribing. Finally, as GPs acknowledged that prescribing support teams did influence their prescribing, they were asked if the interviewer (CFJ) being a prescribing support pharmacist had influenced their responses, to which they indicated that this had not.

Limitations: As GPs were not incentivised to participate we suspect that participants may have been more interested in mental health and psychotropic prescribing, and more willing to openly share experience and reflect on practice. Some potential participants acknowledged that a lack of time and work pressure prohibited study participation when contacted by telephone. Emergent themes were discussed as part of the interview, however these themes were not overtly checked for trustworthiness with future interviewees. The variety and availability of medicalised and non-medicalised support services did vary within the region which may have influenced prescribing, however GPs acknowledged that these were only one aspect of patient care and support, and such variation in support services will also be the case in other urban regions. Finally, although data collected from one large urban region may also be considered a limitation as rurality and regional variations are associated with prescribing variations [32], the findings are of interest to others working in similar urban practices.

### Comparison and links with literature

Safety and risk management were recurring features of this study. Other studies have highlighted that GPs were confident using SSRIs due to perceived and actual safety benefits when compared with other antidepressants [38, 39]. This study highlights that national safety warnings have changed SSRI prescribing practice, reducing citalopram use and increasing sertraline use which is traditionally prescribed at higher doses [21]. It also highlights prescribers’ preference for low dose mirtazapine or trazodone instead of more risky B&Zs. However as with B&Zs, tolerance develops to antidepressant sedative effects within a short period of time [40, 41].

Experience, training and individual patient characteristics influenced drug choice and use, as reported elsewhere [42, 43], however some GPs indicated that prescribing became more “*idiosyncratic*”, using learned experience and habits to achieve the ‘best care fit’ for individuals. In one systematic review such experience was identified as a barrier to the use of evidence-based medicine in practice [44]. However, in contrast to our study, they did not consider the role of specialist services affecting GPs’ treatment decisions, whether that consisted of shared experience and good working relationships, or conversely, a lack of robust support structures and fractured care [45]. In part, some of these idiosyncrasies and experiences may contribute to variations in antidepressant prescribing as one partner within a practice can skew the prescribing figures [46].

Unexpectedly, prescriber preference for waiting as long as 8 to 12 weeks before increasing or switching antidepressants links with a previous quantitative study demonstrating an 8 week lag in drug optimisation [47]. Together these identify a new potential factor which may influence early antidepressant discontinuation, possibly linking with perceived inertia and service dissatisfaction as previously identified [48, 49]. Prescribers also demonstrated that antidepressants were only one of many treatment modalities, and that the GPs themselves had a therapeutic function as listener, counsellor and facilitator [49–51], as well as creating space through use of sickness certificates [52]. As with other studies, GPs rarely saw depression diagnosis and management as a simple task or process. This was due to the complex interplay of social, environmental and comorbidity issues, as well as individuals’ expectations of happiness and unvoiced agendas [38, 53]. Although GPs did not readily take the perceived easy option to prescribe antidepressants, preferring instead to ‘watch and wait’, they would consider prescribing earlier if depressive symptoms were more severe and/or they knew the patient well [42, 50]. Unlike other studies identifying patients’ expectations of antidepressants being a ‘quick fix’ [38, 49], GPs in this study were clear in viewing this as not being unique to depression treatment, but reflecting wider societal expectations.

### Implications for practice

The main implications for practitioners are to be more aware of SSRI dose limitations; higher doses lacking greater efficacy, and time to effect when treating depression [17, 30, 31]. This would potentially have direct and indirect effects on reducing unnecessary prescribing and adverse drug effects, e.g. anxiety and insomnia which might be mistaken for residual depressive symptoms. As well as reducing the co-prescribing of sedating antidepressants, and antipsychotics or B&Zs with their respective cardiometabolic [54] and mood lowering effects [55, 56]. Another dosing concern is the routine use of subtherapeutic mirtazapine doses which may help anxiety and/or insomnia symptoms in the short-term for individuals with mixed depression anxiety but does not optimise depression treatment in line with current evidence [57].

An 8-12 week delaying in drug optimisation potentially slows patient recovery, and when doses and/or drugs are changed it might be harder to differentiate true antidepressant response from spontaneous remission, as 50% of patients recover within 12 weeks [58]. Therefore, in order appropriately optimise antidepressant use for depression treatment it is important that prescribers are more aware that the greatest response occurs within the first 2 weeks of treatment [17, 31].

Finally, a major challenge is creating time to proactively review patients and their antidepressants when individuals are not in a crisis and expecting “*something to be done*”. This is easier said than done, as service pressures, demands and staffing issues limit capacity for proactive reviews. However, where there are safety concerns e.g. MHRA citalopram/escitalopram warning, or as part of a local initiative [21], unnecessary treatment can be reduced or stopped through proactive review.

### Future research

Treatment decisions involving complex ethical and moral processes and judgements to achieve the ‘right care fit’ and to ‘to do the right thing’ for patients which may also be relevant to other areas of prescribing such as antibiotic use and minimisation. Future studies should consider the patients’ perspective and expectations about depression treatment and management and drug limitations. The greater use of antipsychotics, with their known detrimental metabolic effects, to treat depression and/or concomitant insomnia and anxiety is also an area for concern with significant resource implications. Therefore, future studies should consider monitoring and assessing the short and long-term impact of such strategies in primary care.

## Conclusion

GPs strive to ‘do the right thing’ to help people. Antidepressants are only a single facet of depression treatment. However, increased awareness of drug limitations, time to effect, more rapid switching and regular proactive reviews may help optimise care and appropriately minimise antidepressant prescribing.

## Additional file

Additional file 1: Topic guide for first interview (v6) and final version of topic guide (v14). (DOC 43 kb) 
Additional file 2: Supplementary quotes. (DOCX 17 kb)

## Abbreviations

ADM: Antidepressant medicines
B&Zs: Benzodiazepines and/or z-hypnotics
CMHT: Community Mental Health Teams
DDD: Defined daily dose
DNA: Do not attend
GP: General practitioner
MAOI: Monoamine oxidase inhibitor
MHRA: Medicines and Healthcare products Regulatory Agency
NHSGG&C: NHS Greater Glasgow and Clyde
PST: Prescribing support team
SIMD: Scottish Index of Multiple Deprivation
SSRI: Selective serotonin re-uptake inhibitor
TCA: Tricyclic antidepressant
