# Supplementary material for: ‘Doing the right thing’: factors influencing GP prescribing of antidepressants and prescribed doses
Source: BMC Fam Pract. 2017 Jun 17;18:72. doi: 10.1186/s12875-017-0643-z (PMC5473964; doi:10.1186/s12875-017-0643-z)
Supplement: Additional file 1: — Topic guide for first interview (v6) and final version of topic guide (v14). (DOC 43 kb) [file 12875_2017_643_MOESM1_ESM.doc]

**Introductory Questions and Topic Guide – V.6**

**Exploration of factors influencing antidepressant prescribing and prescribed dose: a qualitative study**

**Research question:** What influences prescribers’ use of specific antidepressant and doses?

**Interview questions:**

1. Currently, what would you say are the most important factors influencing antidepressant prescribing and use for the treatment of depression in general practice?
2. How does your **experience** of general practice influence the use of antidepressants?
3. Do **service users** influence your antidepressant prescribing?
4. Does **psychiatry** influence your antidepressant prescribing? How?
5. How do you think your **training** affects your antidepressant prescribing?
6. Does the formulary influence your antidepressant prescribing? How?
7. Has the FDA/MHRA warnings influenced your citalopram/escitalopram prescribing?
8. Generally, what differences are there between antidepressants?
9. How long would you wait for an antidepressant effect when treating depression?
10. What are the therapeutic doses of SSRIs, TCAs, venlafaxine, and mirtazapine?
11. Are there any differences in dose response between SSRIs, TCAs, venlafaxine or mirtazapine for depression?
12. Do you initiate combination antidepressants?
13. When would you initiate combinations antidepressant?
14. Do you think co-prescribing of anxiolytics or hypnotics influences antidepressant response?
15. From our previous work we have found that people prescribed long-term (eg >2 years) antidepressant for depression are prescribed 20% larger doses. SSRIs made up 73% of all antidepressants. From your experience why do you think this may be?

Interview schedule

Version 6, 22/11/13

**Introductory Questions and Topic Guide – V.14**

**Exploration of factors influencing antidepressant prescribing and prescribed dose: a qualitative study**

**Research question:** What influences prescribers’ use of specific antidepressant and doses?

Age Gender How long since qualified? How long as a GP?

Psych rotation? Extra specialist psych training/experience Training practice?

Contract: GMS / 17C

**Interview questions:**

1. In general what factors are contributing to current antidepressant growth?
2. Factors influencing antidepressants use for depression in general practice?

| Service users | Training | | Experience | | Guidelines |
| --- | --- | --- | --- | --- | --- |
| GP colleagues within | Out with your practice | | Psychiatry/CMHTs | | Talking therapies |
| Press/media | | Prescribing Support | | Industry/Pharma | |

1. **What are the challenges with diagnosing depression?**
2. **What are we trying to achieve with antidepressant use?**
3. **How do you qualify and quantify improvement?**
4. Do you think your prescribing is the same as your colleagues? Same, different, normal?
5. **Does gender influence prescribing? GP gender and/or patients?**
6. Policy effects: MHRA citalopram/escitalopram warning (alternatives?)

Formulary Prescribing Indicators

1. Drug effects

| Differences: SSRIs, TCAs SNRIs etc | Response |
| --- | --- |
| Time pressures and decision ↑/↓/cont | Therapeutic doses ?differences |
| Do you Rx combination ADMs | When? |

1. **Guidelines: Able to access information for special groups: pregnant/breast feeding?**
2. How do you think co-prescribing of anxiolytics or hypnotics influences ADM response?
3. From our previous work we have found:
   1. **Practice patients** attended significantly affected the size of SSRI doses
   2. Same SSRI was prescribed for **≥2 years** the doses were significantly larger.
   3. Patients with **B&Zs** prescribed had significantly higher doses.
4. Did me being a PSP influence your answers?

Interview schedule, Version 14, 7/12/15
