# Supplementary material for: ‘Doing the right thing’: factors influencing GP prescribing of antidepressants and prescribed doses
Source: BMC Fam Pract. 2017 Jun 17;18:72. doi: 10.1186/s12875-017-0643-z (PMC5473964; doi:10.1186/s12875-017-0643-z)
Supplement: Additional file 2: — Supplementary quotes. (DOCX 17 kb) [file 12875_2017_643_MOESM2_ESM.docx]

**‘Doing the right thing’: Factors influencing GP prescribing of antidepressants and prescribed doses**

**Supplementary Quotes**

***More than drugs***

*I saw [a woman] a week ago, she has had depression in the past but it was years and years ago. She came in last week. She was in tears. Her mood was low. She’s not sleeping. Classical symptoms of recurrence of her depression. Really stressed at work, changes in her relationships… So, actually I said, “Take the week off work and come back and let’s speak about it. I’d like to take the pressure off there.” Again, she’s not wanting to go onto antidepressants. She’s quite able to make that decision at the moment. She’s seeking counselling and she’s coming back here for regular review and that’s fine. D23,24p1*

**Patients’ expectations and characteristics**

*Undoubtedly. If you're rushed and busy and running and late and the patient comes in looking for an antidepressant, and you've already kept them waiting 40 minutes. It’s very, very hard to sit there and talk about, lifestyle advice and things that they really don't want to hear, that involves a lot of effort on their part. D5,92*

*But yeah, I mean, people will often expect something... that's got historical factors, you know, the prescription from the doctor ‘the bottle’ as they used to call it in Glasgow, you know, your ‘badge’ of your illness, your piece of paper. So going out of the surgery without anything is sometimes, regard as, you know, “sorry for wasting your time doctor,” is often what you get, if you don't actually prescribe something. Or, you hear them talking to the relatives in the waiting area as they leave, “what did he give you?” not, what did he say, “what did he give you?” you know. So there is definitely a societal expectation of a medicine, but you know, most of our patients, you can deal with that. D26,11*

**GP experience and relationships**

*Well, I think as a trainee, [a] very young doc… You perhaps have more influence from your... trainers and from people you've worked with in the practice you're in. Then you go and you work more independently... whether it’s the same practice or whether it’s another practice. You probably start to formulate your own ideas about what’s right... and that may come from evidence; that may come anecdotally. You'll probably try and get a bit of both, you find out what works for you and also what works for patients and also what the guidance would suggest. D4,106*

*…if I trust specialists I've got to know over the years, so [First name psychiatrist], [named Cardiologist] in cardiology, [Named Gastroenterologist] in gastroenterology, [Named consultant] in geriatrics, if [First name geriatrics consultant] say’s give them this, ‘I go that's fine, no problem.’ And if he says I think that's best drug, I am sure it's the best drug. And I actually don't question it. D21,44p2. [although where combination quetiapine and antidepressant was advised by specialist] I might have thought about it, but I will phone up. That the sort of thing I would phone [psychiatrist] about and say, “[psychiatrist], what do you think?” D21,132*

**Antidepressant use: safety, risk management and effective**

***Drug choice***

*My two hot choices at the moment are fluoxetine for true depressive symptoms, and sertraline for depression with anxiety with predominant symptoms. It was citalopram until the guidance regarding prolonged QT. So I tend to go now with sertraline. D27,58*

*I think probably as people try hard to avoid using anxiolytic medication, antidepressants are being used perhaps more for people who don’t have a significant depressive illness but have a bit of depressive/anxiety features. D18,2*

*I think also... you've always got in the back of your mind what you can defend if it all goes wrong. So medical legal issues would worry me a wee bit as well. And another thing is that if someone is using two antidepressants I think you've got to question whether or not they should be seeing a psychiatrist anyway. D22,131*

*I was just thinking there are a couple of people who have just started on quetiapine, as well as an antidepressant. But that’s not off my bat either. D21,132*

***SSRI efficacy: time to effect and dose response***

*Because again I tend to find that after another 4 weeks [8 weeks in total for non-responders] there is a benefit and I don’t really like just chopping and changing antidepressants really quickly, because I think it does take time sometimes, and I don’t like to write them all off too quickly and chop and change people. And then I think you then run out of options too quickly. And again I would maybe encourage people with other things as well. D14,92*

*Yeah, especially for people who are maybe very busy and are in full-time work with a family. They just don't feel that they've got the time to...[engage with other treatment options] Even when you go through the fact that, perhaps the outcome would be better, and a tablet is not going to fix the rest of their issues. They're still going to try it in the hope that they might do something for them. D10,22*

***SSRI efficacy: use of higher SSRI doses for depression treatment***

*I’ll be what they would probably call ‘bad’ - terribly awful. I am sure I over-prescribe antibiotics, over-prescribe benzo, and over-prescribe everything. Over refer... I think some of that, I think sometimes it comes out of a... feeling of wanting to try and do something. I think that's a big internal driver. D21,18p1*

*These are folks that tend to have a bit of resistant anxiety and depression. They've taken a lot of support, and cranking up the doses to get a bit better. And there's a huge resistance, usually, on their part, to bring it down, because of their fear of relapse. So I tend to find the folks that are on higher doses have had really tough times. It's got better, and they're happy where they're at, they're willing to just keep going on that. D27,164*
